# Brain multimodal co-alterations related to delay discounting: a multimodal MRI fusion analysis in persons with and without cocaine use disorder

**DOI:** 10.1186/s12868-021-00654-z

**Published:** 2021-08-20

**Authors:** Christina S. Meade, Xiang Li, Sheri L. Towe, Ryan P. Bell, Vince D. Calhoun, Jing Sui

**Affiliations:** 1 Department of Psychiatry and Behavioral Sciences, Duke University School of Medicine, Box 102848, Durham, NC 27708 USA; 2 Brain Imaging and Analysis Center, Duke University, Durham, NC USA; 3 Brainnetome Center and National Laboratory of Pattern Recognition, Institute of Automation, Chinese Academy of Sciences, Beijing, China; 4 School of Artificial Intelligence, University of Chinese Academy of Sciences, Beijing, China; 5 Tri-Institutional Center for Translational Research in Neuroimaging and Data Science (TReNDS), Georgia State University, Georgia Institute of Technology, Atlanta, GA USA

**Keywords:** Cocaine, Drug addiction, Impulsivity, Delay discounting, Magnetic resonance imaging, Multimodal fusion

## Abstract

**Background:**

Delay discounting has been proposed as a behavioral marker of substance use disorders. Innovative analytic approaches that integrate information from multiple neuroimaging modalities can provide new insights into the complex effects of drug use on the brain. This study implemented a supervised multimodal fusion approach to reveal neural networks associated with delay discounting that distinguish persons with and without cocaine use disorder (CUD).

**Methods:**

Adults with (n = 35) and without (n = 37) CUD completed a magnetic resonance imaging (MRI) scan to acquire high-resolution anatomical, resting-state functional, and diffusion-weighted images. Pre-computed features from each data modality included whole-brain voxel-wise maps for gray matter volume, fractional anisotropy, and regional homogeneity, respectively. With delay discounting as the reference, multimodal canonical component analysis plus joint independent component analysis was used to identify co-alterations in brain structure and function.

**Results:**

The sample was 58% male and 78% African–American. As expected, participants with CUD had higher delay discounting compared to those without CUD. One joint component was identified that correlated with delay discounting across all modalities, involving regions in the thalamus, dorsal striatum, frontopolar cortex, occipital lobe, and corpus callosum. The components were negatively correlated with delay discounting, such that weaker loadings were associated with higher discounting. The component loadings were lower in persons with CUD, meaning the component was expressed less strongly.

**Conclusions:**

Our findings reveal structural and functional co-alterations linked to delay discounting, particularly in brain regions involved in reward salience, executive control, and visual attention and connecting white matter tracts. Importantly, these multimodal networks were weaker in persons with CUD, indicating less cognitive control that may contribute to impulsive behaviors.

## Introduction

Cocaine use continues to be a significant global health problem. In the United States, an estimated 5.5 million people used cocaine in 2018, and nearly 1 million people had a cocaine use disorder (CUD) [1]. Substance use disorders are characterized by persistent neurobiological changes in brain networks that regulate reward salience, decision making, and inhibitory control [2–4]. As substance use progresses to addiction, mesostriatal networks become sensitized to drug cues and desensitized to non-drug rewards [5, 6], while cognitive control networks become hijacked by drug reinforcement [7, 8]. CUD is also associated with deficits in reward-based decision making that are often associated with impulsive behaviors [9], and which may contribute to adverse outcomes, such as infectious disease, criminal behaviors, and violence [10, 11].

Delay discounting, the universal tendency to devalue rewards that are delayed in time [12, 13], has been proposed as a behavioral marker of drug addiction [14]. Delay discounting is a complex cognitive process that involves the mental representation of two reward options with different delays and a subjective evaluation of their values relative to one another [15]. Persons who use drugs consistently demonstrate steeper discounting compared to those who do not use drugs, with the largest effects observed for cocaine and other stimulants [16]. This exaggerated preference for smaller, immediate rewards over larger, delayed rewards has been linked to all stages of drug addiction from initiation of use to addiction severity and treatment outcome [17–19]. Steeper discounting is also predictive of other maladaptive behaviors, such as gambling, risky sex, and overeating [20–22], suggesting that it may represent a trans-disease process [23]. Multiple brain regions across several large-scale neural networks are implicated in delay discounting, likely involving recursive interactions across networks [24, 25]. In non-clinical samples, brain morphology has also been linked to steeper discounting, including lower cortical and higher subcortical gray matter volume (GMV) [26, 27], reduced white mater integrity in brainstem, association, and commissural tracts [28], and lower white matter connectivity between frontal-striatal regions [29]. These neural underpinnings of delay discounting overlap with functional and structural brain alterations identified in CUD.

Magnetic resonance imaging (MRI) studies have provided insight into the neuroanatomical substrates of cocaine and other drug use disorders. Structural MRI studies that quantify gray matter morphology generally find that persons who use cocaine have lower GMV in prefrontal cortices relative to controls, with less consistent reductions in temporal, hippocampal, and cerebellar regions [30–36], along with increased striatal GMV [32]. Resting-state functional MRI (rs-fMRI) studies, which measure the temporal correlation of spontaneous changes in blood flow across spatially distributed regions to identify functional networks, have reported aberrant connectivity in dopamine-rich limbic and subcortical regions involved in reward processing and associative learning, and in fronto-parietal cortical regions that control executive function [37]. Diffusion-weighted imaging (DWI), which characterizes white matter integrity by mapping the movement of water molecules, has shown notable reductions in fractional anisotropy (FA) in the corpus callosum and frontal fiber tracts in persons with CUD compared to controls [38, 39].

While MRI studies have generated vital insights into the long-term effects of chronic cocaine use on the brain, this literature has been dominated by unimodal analyses. Each type of MRI provides unique information on the neural basis of neuropsychiatric disease. Innovative MRI fusion approaches that integrate the information from multiple imaging modalities can unify disparate findings from unimodal analyses, revealing covariation across imaging and clinical measures [40]. Specifically, MCCAR + jICA (multi-site canonical correlation analysis with reference + joint independent component analysis) is a supervised fusion technique that maximizes correlations between the identified brain regions with a reference of interest [41], which might be missed by blind fusion approaches [42–44]. While MCCAR + jICA has been utilized to examine cognitive dysfunction related to a range of psychiatric disorders, such as schizophrenia, major depression, and autism [45–48], this analytic strategy has not yet been used to investigate co-varying functional and structural brain alterations associated with CUD or other substance use disorders.

This study aimed to identify multimodal networks linked to delay discounting that distinguish persons with and without CUD. Existing fMRI studies demonstrate that differences in neural activation and functional connectivity in persons with addictive disorders relate to delay discounting, but there have been too few studies to draw conclusions related to brain structure [49]. Moreover, no studies to date have been designed to identify co-variations in functional and structural brain systems in CUD. To achieve this goal, we implemented a supervised 3-way MRI fusion analysis with delay discounting as the reference. We expected to identify multimodal components comprised of regions implicated in reward processing and cognitive control, and we hypothesized that persons with CUD would have weaker component loadings indicative of alterations in these brain systems.

## Methods and materials

### Sampling

We collected data as part of three protocols that investigated the effects of substance abuse and HIV infection on neural activation during decision making tasks [50–52]. These protocols had shared procedures to facilitate data harmonization. The present analysis includes 72 HIV-negative adults aged 28–55 years who currently used cocaine (COC+) or had no history of cocaine abuse (COC-). The eligibility criteria have been described elsewhere [36, 53]. Participants in the COC + group met the following criteria: current cocaine dependence, regular cocaine use for ≥ 1 year, recurrent cocaine use in the past 30 days, and cocaine as a principal drug of abuse. Participants in the COC− group met the following criteria: no lifetime CUD (abuse or dependence), no history of regular cocaine use, no cocaine use in the past year, and a cocaine-negative drug screen. Alcohol, marijuana, and nicotine use were permitted in all groups. For other drugs, individuals were excluded for any history of dependence, lifetime regular use for > 2 years, regular use in the past year, and any use in the past 30 days. Additional exclusion criteria were: English non-fluency or illiteracy; < 8th grade education; severe learning disability; unresolved neurological disorders or history of neuroinfections; severe head trauma with loss of consciousness > 30 min and persistent functional decline; lifetime bipolar I or psychotic disorder; acute psychiatric symptoms interfering with functioning; MRI contraindications; and/or impaired mental status.

### Procedures

The sample was recruited through posted advertisements in local publications and flyers in nonprofit organizations in Durham, North Carolina and the surrounding area. After a brief telephone interview to assess preliminary eligibility (e.g., no clear MRI contraindication), individuals completed a formal eligibility screening that assessed medical, psychiatric, and substance abuse histories. Participants provided written informed consent prior to enrollment. A rapid HIV test (OraSure ADVANCE^®^ HIV-1/2) was conducted as part of the screen; all participants in this analysis had a non-reactive result. Eligible participants then completed an MRI brain scan and additional assessments.

### Screening measures

The screening visit included several structured clinical interviews. The Addiction Severity Index-Lite assessed functioning across multiple domains, including substance use, psychiatric status, and medical history [54]. The Mini International Neuropsychiatric Interview assessed DSM-IV mood and psychotic disorders [55], while Module E of the Structured Clinical Interview for DSM-IV assessed substance use disorders [56]. An onsite urine toxicology screen was used to identify recent use of amphetamine, barbiturates, benzodiazepines, cannabis, cocaine, methadone, methamphetamine, opioids, and oxycodone. Prior to MRI scan, all participants of childbearing potential also had a urine pregnancy test to ensure MRI safety. The study team also reviewed medical records to verify the absence of any exclusionary substance abuse, psychiatric, or medical conditions.

### Behavioral measures

#### Substance abuse

On the day of the MRI, participants had to have a blood alcohol level of 0.00. Timeline follow-back methodology was used to capture past 90-day use of cocaine and other substances [57]. Another urine toxicology screen was used to assess for presence of cocaine, cannabis, methamphetamine, opioid, and benzodiazepine metabolites. Cocaine craving was measured a 3-item scale that utilizes a visual analog scale [58].

#### Delayed reward discounting

Participants completed the 36-item version of the Monetary Choice Questionnaire (MCQ) [59, 60]. Participants are asked to choose between a series of smaller, immediate rewards ($7–80) and larger, delayed rewards ($7–80 at delays of 1–186 days). The fixed set of items was presented in random order across participants. The task was programed using ePrime (Psychology Software Tools, Inc.; http://www.pstnet.com). Standard scoring procedures were used to compute k-values (MCQ scores), which ranged from 0.00016 to 4.00 [60]. Prior to analyses, the MCQ scores were natural log transformed.

### MRI data acquisition and processing

The MRI data acquisition and processing protocols have been described in detail in a prior report [53]. All scans were conducted on a single 3T GE Discovery MR750 scanner and an 8-channel head coil. In brief, high-resolution T1-weighted (T1w) images were recorded using a spoiled echo sequence (1 mm^3^ voxels, 1 mm interleaved slices). Diffusion-weighted images (DWI) were acquired in the axial plane using a diffusion sensitized parallel echo-planar sequence (2 mm^3^ voxel size, 2 mm interleaved slices), with 30 diffusion-encoding directions included in analyses. Finally, whole-brain blood oxygenation level dependent (BOLD) images were collected while participants fixated on a crosshair using T2*-weighted echo-planar imaging (3.75 × 3.75 × 3.8 mm voxel size, 3.8 mm interleaved slices), with 148 volumes included. All analyses included protocol as a covariate of no interest.

Pre-processing was primarily implemented in FMRIB Software Library (FSL) version 5.0.9 [61]. The T1w images were pre-processed using standard methods [62, 63], and then participant-level maps of GMV were created using voxel-based morphometry [64, 65]. The DWI data were denoised [66], motion and eddy-corrected using DTIPrep [67], and then preprocessed using standard FSL tools [61]. FA was calculated within all white matter voxels. The rs-fMRI data were preprocessed and denoised using a standard pipeline in FSL [63, 68, 69]. Regional homogeneity (ReHo), which assesses the similarity of the fMRI timeseries for each voxel to its 3-dimensional 27-voxel neighborhood, was calculated using the AFNI tool 3dReHo [70]. ReHo, a voxel-based measure of neural activity, uses Kendall’s coefficient of concordance to identify functional clusters with synchronized timeseries [71].

### Multimodal data fusion

Images were re-sampled to 3 mm and spatially smoothed using a Gaussian kernel with a full width at half maximum of 6 mm. The three-dimensional images of each participant were reshaped into a 1-dimensional vector and stacked, forming a matrix (${\mathrm{N}}_{\mathrm{participant}}\times {\mathrm{N}}_{\mathrm{voxel}}$) for each modality. These matrices were normalized to have the same average sum-of-squares (computed across participants and voxels). Multivariate analysis of covariance (MANCOVA) was performed on the feature of each voxel to regress out the potential effects of age, gender, and protocol.

The preprocessed MRI features were fed into the MCCAR + jICA pipeline, a data-driven fusion method (Fusion ICA Toolbox; http://trendscenter.org/software/fit), as described previously [53]. MCQ scores were entered as the a priori reference measure, and analyses were conducted agnostic to group label. The MCCAR analysis resulted in canonical variants (CVs) that were most correlated across participants between modalities. The joint ICA was then applied to the concatenated spatial maps of all CVs using the Infomax algorithm to retain modality linkage while maximizing the spatial independence of the components. Using the minimum description length criterion [72], 17 components were estimated with corresponding participant-wise loadings derived from each modality. We defined independent components (ICs) from the same index across all three modalities as joint ICs. Pearson correlations were used to examine the strength of the relationship between the component loadings and the MCQ score. MANOVA was used to compare the COC+ and COC− groups on the component loadings. The final joint ICs were selected based on both: (1) loadings that correlated significantly with the reference across all three modalities at p < 0.05, and (2) group differences between COC+ and COC− were significant at p < 0.05 in at least one modality. The brain regions contributing to the joint IC were identified using the Harvard–Oxford Atlas for GMV and ReHo and the IIT Human Brain Atlas for FA [73, 74]. In secondary analyses, Pearson correlations and independent samples t-tests were used to examine the strength of the relationship between the component loadings and cocaine-related clinical measures.

## Results

### Participant characteristics

The sample of 35 COC + and 37 COC− participants was predominantly male (58%) and African–American (78%) with a mean age of 44.18 years (SD = 7.06) (Table 1). The groups were well matched on gender, race, and age, but COC + had significantly fewer years of education than COC−. Participants in both groups reported past month use of nicotine (50%), alcohol (69%), and marijuana (36%), but COC+ were significantly more likely to have used nicotine and alcohol.

**Table 1:** Sample characteristics by COC group

	COC+ N = 35	COC− N = 37	Statistic	p-value
Demographics				
Age in years, M (SD)	45.40 (6.45)	43.03 (7.50)	t(70) = 1.44	0.156
Male gender, n (%)	21 (60%)	21 (57%)	χ(1)^2^ = 0.08	0.780
African-American race, n (%)	28 (80%)	28(76%)	χ(1)^2^ = 0.20	0.659
Education in years, M (SD)	12.54 (2.58)	13.97 (2.03)	t(70) = 2.62	0.011
Other substance use in past 30 days				
Daily nicotine, n (%)	21 (60%)	12 (32%)	χ(1)^2^ = 5.51	0.019
Any alcohol, n (%)	30 (86%)	20 (54%)	χ(1)^2^ = 8.50	0.004
Any marijuana, n (%)	16 (46%)	10 (27%)	χ(1)^2^ = 2.72	0.099
Number of substances used, M (SD)	2.00 (0.77)	1.14 (1.06)	t(70) = 3.95	< 0.001

Participants in the COC + group reported using cocaine regularly for longer than a decade (M = 17.43 years, SD = 8.43). They had used cocaine an average of 9.94 days (SD = 6.39) in the 30 days prior to the screening visit, and smoking was by far the most common primary route of administration (92%). On the day of the scan, the majority (79%) tested positive for cocaine on the urine toxicology screen and reported use within the past 3 days (74%). The median number of days since last use was 2 (IQR = 1,4).

### Performance on the monetary choice questionnaire

As expected, MCQ scores (natural log transformed) were significantly higher in the COC + group (M = − 2.46, SD = 1.19) compared to the COC− group (M = − 3.63, SD = 1.79; t(70) = 3.25, p = 0.002), indicating that they discount delayed rewards more steeply. In monetary terms, when considering a delayed reward of $50 in 7 days, the average COC− participant was approximately indifferent when the immediate reward was $31, whereas the average COC + participant was approximately indifferent when the immediate reward was $25.

### Group-discriminating joint components

One joint component (IC_6_) was correlated with MCQ score across all three modalities. The component loadings for each modality were negatively correlated with MCQ scores, indicating that higher loadings were associated with lower delay discounting. There were significant group differences in the component loadings of all three modalities [F (3,68) = 5.10, p = 0.003; Wilk's Λ = 0.82], which were lower in COC+ compared to COC− (Table 2), meaning that the component was expressed less strongly in persons with CUD. Additional components differed across groups, but did not meet the definition of a joint component.

**Table 2:** Group comparison on IC_6_ loadings

	COC− (N = 37)	COC+ (N = 35)	Statistic	p-value
Gray matter volume, M (SD)	0.097 (0.016)	0.088 (0.013)	F(1, 70) = 6.621	0.012
Fractional anisotropy, M (SD)	0.031 (0.016)	0.021 (0.013)	F(1, 70) = 6.957	0.010
Regional homogeneity, M (SD)	− 0.059 (0.015)	− 0.069 (0.015)	F(1, 70) = 9.356	0.003

**Fig. 1:**
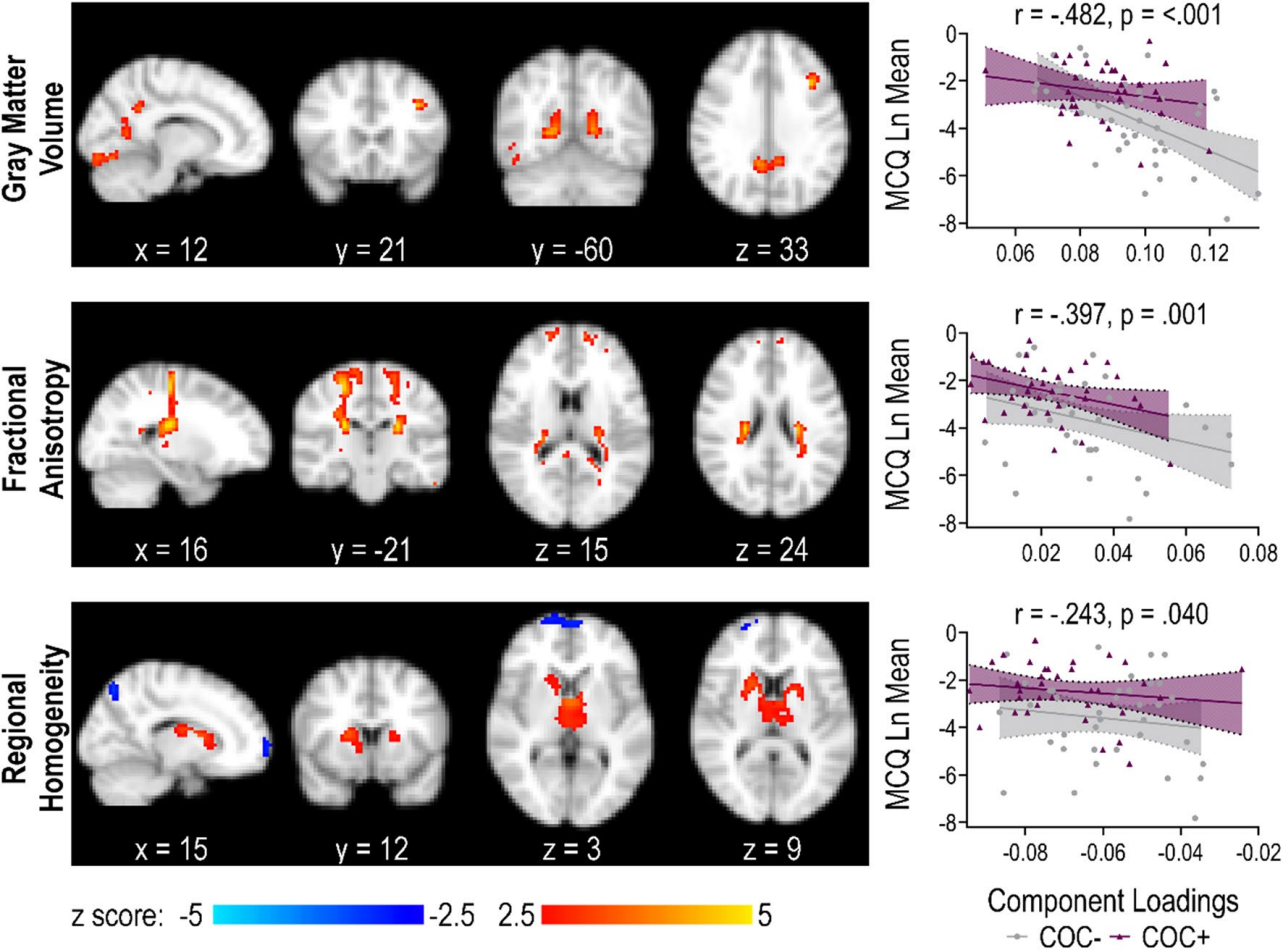
Multimodal group-discriminating IC_6_. For each modality, spatial maps are shown at a threshold of $\left|Z\right|$ ≥ 2.5. For gray matter volume (GMV) and fractional anisotropy (FA), the component loadings are positive. Positive Z-values (red regions) means that COC− have higher GMV and FA compared to COC+. For regional homogeneity (ReHo), the components loadings are negative. Positive Z-values (red regions) means that COC− have lower connectivity compared to COC+, while negative Z-values (blue regions) means that COC− have higher values compared to COC+. The scatter plots on the right show the relationship of the component loadings to delay discounting

Figure 1 displays representative spatial maps for IC_6_. This component was characterized by GMV in bilateral occipital regions (occipital fusiform, occipital pole, intracalcarine and supracalcarine cortices, and lingual gyrus), posterior cingulate cortex, precuneus, and left dorsolateral prefrontal cortex, along with FA throughout bilateral parietopontine, frontopontine, corticospinal, frontal aslant, parietal aslant, superior longitudinal fasciculus, corpus callosum, and arcuate fasciculus. These structural components correlated positively with ReHo in subcortical clusters including thalamus and dorsal striatum and negatively with bilateral frontal pole. Table 3 details the identified clusters.

**Table 3:** Regions in joint IC_6_ at Z ≥|2.5|

Anatomical region(s) in cluster	MNI coordinates at max (x, y, z)	Number of voxels	Max z-score
Gray matter volume			
Positive			
B. occipital fusiform gyrus^a^, lingual gyrus, occipital pole	27, − 78, − 15	277	4.49
L. middle frontal gyrus^a^	− 36, 18, 33	31	4.37
R. precuneus^a^, intracalcarine and supracalcarine cortices, lingual gyrus	18, − 57, 6	87	4.32
L. precuneus^a^, intracalcarine and supracalcarine cortices, lingual gyrus	− 15, − 60, 9	97	3.92
B. precuneus^a^, posterior cingulate cortex	9, − 51, 33	146	3.86
Fractional anisotropy			
Positive			
R. parietopontine^a^, frontopontine, corticospinal, frontal aslant, arcuate fasciculus, corpus callosum (body and splenium), superior longitudinal fasciculus	24, − 21, 21	247	4.70
L. frontopontine^a^, parietopontine, corticospinal, frontal aslant, arcuate fasciculus, corpus callosum (body and splenium), superior longitudinal fasciculus	− 24, − 24, 21	142	4.66
L. frontopontine^a^, parietopontine, corticospinal, frontal aslant	− 18, − 24, 60	84	3.61
Regional homogeneity			
Positive			
B. thalamus^a^, caudate, putamen	0, 0, 3	478	3.61
R. inferior lateral occipital cortex^a^, occipital pole	42, − 90, − 9	62	3.19
Negative			
R. superior lateral occipital cortex^a^	18, − 72, 54	35	3.27
B. frontal pole^a^	− 3, 66, 0	120	3.04

^a^Indicates regions at max

Within the COC+ group, we examined the association between IC_6_ component loadings and cocaine-related clinical factors. For each modality, there were no significant correlations for years of regular cocaine use, days of cocaine use in the past 90, and cocaine craving on the day of the MRI. There were also no significant differences in the component loadings based on urine drug screen result for cocaine on the day of the MRI. While the use of nicotine, alcohol, and marijuana was prevalent in both groups, COC+ participants were more likely than COC− participants to be using these substances. The group differences in the component loadings remained significant when controlling for the number of substances used [F (3, 67) = 3.13, p = 0.031; Wilk’s Λ = 0.87], with COC + having lower scores for GMV [F (1, 69) = 4.14, p = 0.046], FA [F (1, 69) = 4.88, p = 0.030], and ReHo [F (1, 69) = 5.09, p = 0.027].

## Discussion

The goal of the present study was to compare persons with and without CUD on multimodal neural patterns linked to the transdiagnostic index of delay discounting. Unlike single modality analyses, multimodal fusion can reveal latent information that covaries across MRI modalities. Using MCCAR + jICA, we identified one joint component that correlated with delay discounting across all three modalities. These components included gray matter nodes involved in reward salience, executive control, and visual attention and white matter tracts connecting these relevant regions. This component was also group discriminating, such that the component scores were lower in persons with CUD compared to those without CUD. Our results support linkages between structural alterations and neuronal function in CUD that may have relevance for impulsive decision making.

The joint component included regions involved in reward valuation and visual processing. Specifically, it was characterized by positive neural activity in the thalamus and dorsal striatum, and negative neural activity in the frontopolar cortex. In non-clinical samples (75, 76), steeper discounting is associated with increased activity in the ventral and dorsal striatum, coupled with reduced activity in the medial prefrontal cortex [77]. The rostral portion of the medial prefrontal cortex is believed to represent delayed rewards [78, 79], while the nucleus accumbens is activated in response to positive subjective value [80]. Recent models of delay discounting suggest that thalamic-cortical circuits are critical for supporting interactions between multiple neural systems [24]. Thus, our identified neuroimaging components extend prior findings from multiple unimodal studies by demonstrating linkages between brain function and structure in relation to delay discounting.

Furthermore, we found that neural activation patterns were linked to GMV broadly throughout primary and extra-striate networks and association areas, essential parts of the visual attention network [81], suggesting that visual processing has its role in reward [82]. It may be that aberrant visual processing in CUD contributes to dysfunctional attentional processing of competing rewards. Specifically, we found that GMV reductions in visual cortex were significantly correlated with higher discounting. This is consistent with a recent analysis from the Human Connectome Project that found higher discounting to be associated with reduced surface area and volume across multiple cortical regions, including bilateral lingual gyrus [28]. Furthermore, this component indicated linkage between GMV and white matter integrity in the splenium of the corpus callosum, parietopontine, frontopontine, and frontal aslant tracts. Alterations in white matter integrity may disrupt the processing of competing options, resulting in higher discounting of delayed rewards. In sum, our results support the relevance of the visual cortex, likely mediated by attentional processing, to delay discounting.

A key finding of our study is that persons with CUD had lower component loadings for all three modalities in the joint component related to delay discounting. This suggests that CUD is associated with hyperactivity in the thalamus and dorsal striatum but hypoactivity in the prefrontal cortex, as well as altered morphology in linked gray matter regions and white matter tracts. Prior studies conducted across different addictions support the relevance of frontal-striatal circuitry to delay discounting, with higher delay discounting being associated with activation in the thalamus and midbrain regions and deactivation in the frontopolar cortex [49, 83]. While alterations in gray and white matter structure are consistently observed in persons with CUD [30, 32, 36], there has been insufficient research to draw conclusions about how these morphological differences relate to delay discounting [49]. The current study suggests that alterations in attentional networks, coupled with reduced integrity in linked white matter tracts, may contribute to the exaggerated discounting often observed in persons with CUD and other addictions.

While the multimodal component associated with delay discounting was expressed more weakly in persons with CUD compared to controls, the component loadings were unrelated to cocaine-related variables. The CUD group was characterized by chronic cocaine use, with a mean of 17 years of regular use, and all participants met criteria for cocaine dependence. Moreover, the sample was defined by current cocaine use, with a relatively high frequency of use in the past month and most having used within 3 days of the MRI scan. The low variability in cocaine characteristics likely limited our ability to identify correlations with the identified component. A larger and more heterogeneous sample is needed to determine the extent to which CUD severity and cumulative cocaine exposure may drive reductions in multimodal brain systems, and longitudinal designs are needed to verify the temporal relationship of cocaine use to structural and functional changes in the brain related to delay discounting.

Despite the innovative analytic approach and insightful results, there are several limitations to highlight. First, while we selected MRI features that reflect complementary views of brain structure and function, different imaging techniques may provide additional perspectives. Future studies might consider the inclusion of alternative features, such as functional network connectivity and task-evoked neural activation. Second, ReHo characterizes the local functional connectivity between a given voxel and its nearest neighbors, which is one possible index for describing the importance of a voxel in a network [84]. While ReHo features tend to be more tractable for interpreting linkage, future studies should examine the contribution of long distance connectivity to delay discounting. Third, our analyses identified only one multimodal component that correlated with delay discounting that differed between groups. It is common for MCCAR + jICA to reveal just one or two components relevant to the construct of interest because MCCAR is optimized to achieve a single component that is most correlated with the reference measure. Finally, given the modest sample size, replication results with an independent dataset is needed to strengthen the interpretability of our findings.

In summary, we applied supervised data fusion to reveal linked structural and functional neural patterns related to delay discounting that distinguished persons with and without CUD. While other methodologies rely on a direct morphologic connection to link brain regions, multimodal fusion is capable of identifying linked alterations in spatially distinct brain regions, which is a major strength of this approach. Building upon unimodal MRI studies, our results reaffirm that delay discounting is a complex cognitive process that involves interactions among multiple structural and functional networks [49]. Importantly, we found that persons with CUD had lower component scores across modalities, suggesting that alterations in functional networks, cortical and subcortical regions, and connecting white matter tracts contribute to the exaggerated discounting that is characteristic of addiction and other psychiatric disorders. This innovative multimodal fusion analysis has the potential to uncover biomarkers of CUD, and an exciting future direction is to incorporate longitudinal multimodal imaging and clinical data into the fusion model. By expanding the toolkits available for CUD research, it may be possible to develop biomarkers capable of diagnostic performance.

## Data Availability

The datasets analyzed for the current study are not publicly available because we did not obtain the consent of participants to provide them to third parties, but the data is available from the corresponding author on reasonable request.
